# Artificial Intelligence in Identifying Patients With Undiagnosed Nonalcoholic Steatohepatitis

**DOI:** 10.36469/001c.123645

**Published:** 2024-09-25

**Authors:** Onur Baser, Gabriela Samayoa, Nehir Yapar, Erdem Baser

**Affiliations:** 1 Graduate School of Public Health, City University of New York, New York, NY, USA; 2 University of Michigan Medical School, Ann Arbor, Michigan, USA; 3 John D. Dingell VA Center, Detroit, Michigan, USA; 4 Columbia Data Analytics, Ann Arbor, Michigan, USA

**Keywords:** data analysis, machine learning, nonalcoholic fatty liver disease, Veterans Health Services

## Abstract

**Background:** Although increasing in prevalence, nonalcoholic steatohepatitis (NASH) is often undiagnosed in clinical practice.

**Objective:** This study identified patients in the Veterans Affairs (VA) health system who likely had undiagnosed NASH using a machine learning algorithm.

**Methods:** From a VA data set of 25 million adult enrollees, the study population was divided into NASH-positive, non-NASH, and at-risk cohorts. We performed a claims data analysis using a machine learning algorithm. To build our model, the study population was randomly divided into an 80% training subset and a 20% testing subset and tested and trained using a cross-validation technique. In addition to the baseline model, a gradient-boosted classification tree, naïve Bayes, and random forest model were created and compared using receiver operator characteristics, area under the curve, and accuracy. The best performing model was retrained on the full 80% training subset and applied to the 20% testing subset to calculate the performance metrics.

**Results:** In total, 4 223 443 patients met the study inclusion criteria, of whom 4903 were positive for NASH and 35 528 were non-NASH patients. The remainder was in the at-risk patient cohort, of which 514 997 patients (12%) were identified as likely to have NASH. Age, obesity, and abnormal liver function tests were the top determinants in assigning NASH probability.

**Conclusions:** Utilization of machine learning to predict NASH allows for wider recognition, timely intervention, and targeted treatments to improve or mitigate disease progression and could be used as an initial screening tool.

## BACKGROUND

Nonalcoholic fatty liver disease (NAFLD) is a significant public health concern, affecting 30% of the adult population in the United States^1,2^ and at least a quarter of the global population.^3^ NAFLD has likely exceeded 30% prevalence in most middle- and high-income countries.^3^ Although NAFLD is strongly linked to obesity and metabolic disorders,^3^ 43% of people with NAFLD in the United States and 71% in Sweden do not have obesity.^3,4^ In the United States, NAFLD is the fastest growing indication for liver transplantation, accounting for more than 25% of these procedures.^3,5^ It is also the fastest-growing cause of liver cancer among candidates for liver transplantation.^3,6^ While the effect of NAFLD on mortality has been masked by cardiovascular disease and extrahepatic malignancies, we can expect to see more people with severe disease dying of liver-related conditions in the future.^3,7^

NAFLD encompasses a spectrum of liver lesions, from steatosis to cirrhosis.^3^ NAFLD is classified histologically as nonalcoholic fatty liver (NAFL) or nonalcoholic steatohepatitis (NASH). NAFL is characterized by fat accumulation (steatosis) in the liver without significant inflammation.^8^ NASH, which is characterized by a liver fat content of more than 5.5%^9^ and inflammation with hepatocyte injury (ballooning) with or without fibrosis, can lead to end-stage liver disease, cirrhosis, or hepatocellular carcinoma.^1,8^ Although NASH is a leading risk factor for end-stage liver disease and cardiometabolic diseases, it often goes undiagnosed in clinical practice because of the need for direct imaging assessments.^9^

Another reason for NASH underdiagnosis is the presentation of asymptomatic or nonspecific symptoms, which may not be overt on clinical examination or routine laboratory tests.^1^ Despite this, NASH prevalence is rapidly increasing alongside the global epidemics of obesity and diabetes.^9^ The current reference standard for diagnosing and staging NASH (steatosis vs steatohepatitis) is liver biopsy, an invasive and costly procedure associated with risks such as postprocedural pain and bleeding.^1,3^ There remains a need to develop a noninvasive tool to identify patients with NASH while ruling out those unlikely to have advanced liver disease, without the need for biopsy.

Previous studies found only 25% of patients with radiographic evidence of liver steatosis received a NAFLD diagnosis.^10^ Among undiagnosed patients, 9% to 17% had high-risk advanced fibrosis scores, suggesting that providers are missing cases of advanced disease.^10^ The diagnosis rate of NASH varies by disease stage, with lower rates of diagnosis in earlier stages. For example, only 2.0% of patients in stages F0 and F1, and 16.5% in stage F2 were diagnosed.^11^ Overall, an estimated 79.8% of the prevalent NASH population in the United Kingdom in 2018 were not diagnosed.^11^ Endocrinologists reported low knowledge of which blood markers to use when suspecting NASH and difficulties interpreting test results, which can contribute to diagnostic challenges.

The use of machine learning (ML) in health care has increased in tandem with electronic health records and the advancement of big data analytics.^1,12^ ML, an area of artificial intelligence, is increasingly being used to address issues affecting patients, payers, and providers. ML is uses computer algorithms that can learn complex patterns from data.^13^ These algorithms can help in acquiring, interpreting, and synthesizing healthcare data from diverse sources.^13^ While ML can be useful in many healthcare contexts, one intriguing application is the prediction of disease presence among individual patients within large databases.

ML is a multidisciplinary subject involving probability theory, statistics, approximation theory, convex analysis, algorithm complexity theory, and other fields.^14^ By continuously acquiring new knowledge, processing information, and reorganizing existing knowledge to improve performance, ML simulates human learning behaviors.^14^ ML is used to improve on traditional risk prediction algorithms with available registry data and test whether advanced insights are possible from data types that go largely unutilized. ML has been used to diagnose fatty liver, meningitis, glaucoma, coronary heart disease, cancer, and other diseases.^15^

Prior work has shown promise for applying ML to predict NASH, but many of these approaches do not use readily available inputs or have not yet been validated in a large cohort.^1^ Identifying patients at risk of NASH in whom steatohepatitis is active and moderate fibrosis is present (NAFLD activity score ≥4, fibrosis stage ≥2) is important in understanding the likelihood of progression to cirrhosis.^3^ Successfully identifying patients at risk of NASH or in the earliest stage of disease can also allow for specific stratification, counseling, and disease management in settings outside specialized clinics, such as primary care facilities. Early identification not only can help prevent the disease from progressing to more serious conditions such as cirrhosis but also could help lower healthcare costs associated with specialized clinics and increase physician awareness of the disease.^1,16^

The purpose of this study was to identify patients in the Veterans Affairs (VA) health system who likely had NASH but had not been diagnosed yet. To identify the risk group, we performed a claims data analysis using ML algorithms, which are increasingly being used as a predictive tool in medicine.

## METHODS

The VA data set used for analysis included 25 million enrollees as of December 2022 and contained inpatient and outpatient files, lab information, survival statistics, and vital statistics, including height, weight, and blood pressure collected from 152 VA hospitals, 133 VA Community Living Centers, and 958 outpatient clinics.

The study population, comprising patients between the ages of 18 and 85 years, was divided into 3 cohorts:

1. The **positive NASH cohort** consisted of the patients who received a NASH diagnosis during the outcome window, defined as the 12 months prior to the latest available date in the data set.
2. The **non-NASH cohort** was defined as the patients who had nonalcoholic fibrosis or cirrhosis during the outcome window but no NASH diagnosis.
3. The **patients-at-risk cohort** was defined as those with at least 1 diagnosis of alcoholic liver disease, liver disorders in pregnancy, primary sclerosing cholangitis, hepatorenal syndrome, portal hypertension, primary biliary cirrhosis, nonalcoholic liver disease, toxic liver disease, spontaneous bacterial peritonitis, ascites, esophageal varices, alcohol abuse, α_1_-antitrypsin deficiency, hemochromatosis, Wilson disease, Gaucher disease, liver cancer, hepatitis, other alcohol-related conditions, or encephalopathy during the baseline window 24 months prior to the index date.

The index date was defined randomly between the minimum and maximum dates of NASH diagnosis from the first cohort. This random selection helps mitigate potential biases that could arise from selecting a specific date, ensuring that the study’s results are not skewed by temporal trends in the data. For the at-risk patient cohort, we extracted all claims from the source data and constructed an enriched study population by selecting individuals who fell into the broader categories of diagnoses that are prone to NASH. Details of each group are shown in **Table 1**.

**Table 1.** Positive NASH, Non-NASH, and At-risk NASH Cohorts

**Cohort Characteristics**	**n**
Positive NASH cohort	
Inclusion	
≥1 diagnosis of NASH during outcome window	8180
Age 18-85 years	8144
Exclusion	
Having ≥1 diagnosis of NASH during 24 months prior to index date (baseline)	4903
Non-NASH cohort	
Inclusion	
≥1 diagnosis of nonalcoholic liver fibrosis or cirrhosis during outcome window	40 753
Age 18-85 years	40 241
Exclusion	
≥1 diagnosis of NASH during 24 months prior to index date (baseline)	35 528
Unknown NASH cohort	
Inclusion	
Age 18-85 years	4 674 355
Exclusion	
≥1 diagnosis of alcoholic liver disease, liver disorders in pregnancy, primary sclerosing cholangitis, hepatorenal syndrome, portal hypertension, primary biliary cirrhosis, nonalcoholic liver disease, toxic liver disease, spontaneous bacterial peritonitis, ascites, esophageal varices, alcohol abuse, α_1_-antitrypsin deficiency, hemochromatosis, Wilson disease, Gaucher disease, liver cancer, hepatitis, other alcohol-related conditions, encephalopathy	4 183 012

Abbreviation: NASH, nonalcoholic steatohepatitis.

The exploratory data analysis revealed patterns and correlations in the data that indicated the feasibility of using machine learning algorithms. For instance, the presence of significant comorbidities like obesity and diabetes, which are strong predictors of NASH, would support the use of ML to identify undiagnosed cases. Therefore, we performed a claims data analysis using an ML algorithm. To build our model, the study population was randomly divided into an 80% training subset and a 20% testing subset based on patient IDs. A cross-validation technique was used to test and train the model on different iterations. The performance metrics were determined by the training subset; after selecting the highest-performing model, the testing subset was used to evaluate the performance of the best-performing ML model. Then, a baseline model (logistic regression) was created to predict the classification of an observation based on several explanatory variables such as demographic and clinical factors; in this case, the observation was whether a patient has NASH. The relevant variables in the VA claims dataset served as the basis for gender (female or male), age (18-34, 35-44, 45-54, 55-64, ≥65 years), and race (white, black, unknown, other). NASH and its associated clinical comorbidities were identified through the utilization of *International Classification of Diseases, 10th Revision* (ICD-10) codes. Specific indicators were established to identify the presence of obesity, type 2 diabetes mellitus, metabolic disorders, NAFLD, and hypertension.^17^ Procedures for diagnosing and managing NASH were identified from claims data. These include liver biopsy, liver panel tests, abnormal liver function tests, detection of abnormal levels of other serum enzymes, abdominal ultrasound, and comprehensive metabolic panels. These diagnostic tools are critical in assessing liver health and guiding the management of NASH.

Additional models, such as a gradient-boosted classification tree, naïve Bayes, and random forest, were also created. The models chosen are well-suited for handling complex data relationships and imbalances, offering robust performance metrics like receiver operator characteristics^18^ and area under the curve (AUC). Gradient-boosted trees often provide higher accuracy by combining multiple weak learners to improve performance, and are effective at capturing complex relationships in data by sequentially correcting errors from previous models and optimizing a variety of loss functions handling missing data without imputation.^19^ Naïve Bayes is suitable for solving multiclass classification problems and works well with large data sets.^20^ Random forests are less prone to overfitting and provide insights into feature importance, aiding in understanding data patterns.^21^ Adjustments were applied to handle 15% “imbalanced data,” which occurs when one or more of the binary classes are underrepresented. These adjustments included modifying class weights, oversampling, and under sampling. A 3 × 3 cross-validation was applied to several random forest models to determine the set of parameters that define the properties of the model, such as the number of decision trees in the random forest.

The performance metrics of all models were examined, and model performance was evaluated using the outputs of each patient’s NASH probability. The metrics used to compare models were ROC, AUC, and accuracy. They provide a comprehensive view of model performance, particularly in imbalanced datasets. ROC and AUC are advantageous as they are less affected by class imbalance, while accuracy provides a straightforward performance measure.^22^ The best-performing model was retrained on the full 80% training subset and applied to the 20% testing subset to calculate the performance metrics. To determine the optimal hyperparameters for the best-performing machine learning models, specific parameters were considered for each model. For gradient-boosted trees, the learning rate was adjusted to control the contribution of each tree to the final model. In the case of naïve Bayes, the smoothing parameter was tuned to regulate the strength of Laplace smoothing. For random forests, the number of trees in the forest and the number of features considered for splitting at each node (typically the square root of the total number of features) were optimized. Results were summarized in a confusion matrix, and an ROC curve was developed.

## RESULTS

A retrospective analysis of the Veterans Affairs (VA) database identified 4 223 443 patients who met the study inclusion criteria. Based on their respective diagnosis codes, patients were stratified into 3 distinct cohorts: NASH-positive (n = 4903), non-NASH (n = 35 528), and at-risk (n = 4 183 012). The NASH-positive cohort comprised patients with confirmed NASH diagnoses, while the non-NASH cohort included patients with liver-related diagnoses other than NASH. The remaining patients, who did not fall into either of these categories but met the study’s inclusion criteria, were classified as the at-risk patient cohort (**Table 1**).

The demographic and clinical profiles of each group are presented in **Table 2**. Compared with the positive-NASH diagnosis group, the non-NASH group was more likely to be older than 65 years (*P* < .0001), male (*P* < .0001), or Black, and less likely to have any comorbidities or procedures. The at-risk group were more likely to be over 65 years old (*P* < .0001) and white (*P* < .0001) and less likely to have any comorbidities or procedures relative to the NASH-positive diagnosis group (*P* < .0001) (**Table 2**). The NASH-positive group was more likely to be older (>65 years) (*P* < .0001), male (93.8%), and white (77.95%), and to have comorbidities. The most common comorbidities were type 2 diabetes (50.58%), and obesity (44.40%). Moreover, 89% of patients had a liver panel test performed, 31.53% had abnormal liver function test, 51.48% had an abdominal ultrasound, and 3.96% had undergone a liver biopsy.

**Table 2.** Demographics and Clinical Profiles of Each NASH Cohort Group

**Characteristics**	**NASH (n = 4903)**	**Non-NASH (n = 35,528)**	**At Risk of NASH (n = 4,183,012)**	***P* Value**	
				**NASH vs Non-NASH**	**NASH vs At Risk of NASH**
Age, years					
Mean (SD^a^/SE^b^)	58.54 (12.82^a^)	64.31 (7.23^a^)	61.12 (15.24^b^)	<.0001	<.0001
18-34, n (%)	284 (5.79)	82 (0.23)	362 065 (8.66)	<.0001	<.0001
35-44, n (%)	503 (10.26)	325 (0.91)	333 367 (7.97)	<.0001	<.0001
45-54, n (%)	842 (17.17)	1959 (5.51)	517 268 (12.37)	<.0001	<.0001
55-64, n (%)	1 213 (24.74)	15 358 (43.23)	773 434 (18.49)	<.0001	<.0001
≥65, n (%)	2 061 (42.04)	17 804 (50.11)	2 196 878 (52.52)	<.0001	<.0001
Gender, n (%)					
Male	4550 (92.80)	34 474 (97.03)	3 839 218 (91.78)	<.0001	.0094
Race, n (%)					
White	3822 (77.95)	23 596 (66.42)	2 928 774 (70.02)	<.0001	<.0001
Black	503 (10.26)	8466 (23.83)	665 163 (15.90)	<.0001	<.0001
Unknown	370 (7.55)	2428 (6.83)	457 766 (10.94)	.0654	<.0001
Other	208 (4.24)	1038 (2.92)	131 309 (3.14)	<.0001	<.0001
Comorbidities, n (%)					
Obesity	2177 (44.40)	7143 (20.11)	760 739 (18.19)	<.0001	<.0001
Type 2 diabetes	2480 (50.58)	14 474 (40.74)	1 051 137 (25.13)	<.0001	<.0001
Metabolic disorder	2 (0.04)	16 (0.05)	715 (0.02)	.8950	.2050
NAFL	751 (15.32)	1392 (3.92)	10 479 (0.25)	<.0001	<.0001
Hypertension	3358 (68.49)	24 975 (70.30)	2 220 271 (53.08)	0.0096	<.0001
Procedures, n (%)					
Liver biopsy	194 (3.96)	796 (2.24)	910 (0.02)	<.0001	<.0001
Liver panel	4364 (89.01)	28 352 (79.80)	3 044 204 (72.78)	<.0001	<.0001
Abnormal liver function test	1546 (31.53)	4344 (12.23)	74 539 (1.78)	<.0001	<.0001
Abnormal levels of other serum enzymes	316 (6.45)	1050 (2.96)	20 412 (0.49)	<.0001	<.0001
Abdominal ultrasound	2524 (51.48)	20 930 (58.91)	141 171 (3.37)	<.0001	<.0001
Comprehensive metabolic panel	3271 (66.71)	23 537 (66.25)	2 165 517 (51.77)	.5184	<.0001

^a^Standard deviation (SD).

^b^Standard error.

Abbreviations: NAFL, nonalcoholic fatty liver; NASH, nonalcoholic steatohepatitis; SE, standard error.

**Figure 1.**
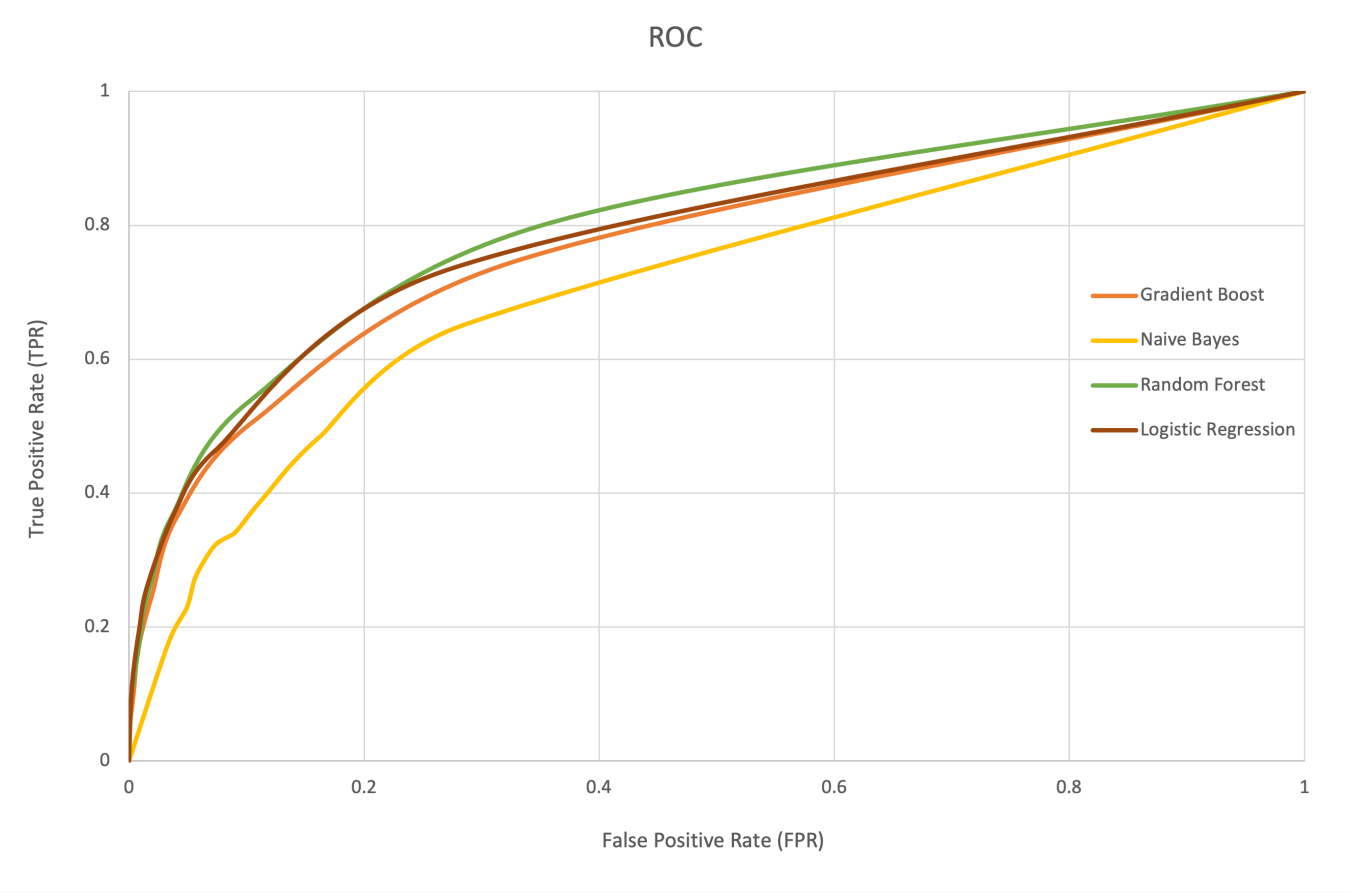
ROC Curves for the Models Source: Data from present study. Abbreviation: ROC, receiver operator characteristics.

Since true positives and true negatives were underrepresented, the data were considered imbalanced. Additionally, of the 4 models applied, random forest contained the highest scores (**Table 3**). The ROC curve denotes the performance of the classification models at all classification thresholds (**Figure 1)**. This curve plots 2 parameters: true-positive rate and false-positive rate. The green curve (random forest model) covers slightly more area; thus, the AUC was highest with that model and obtained better results. By contrast, the worst results were obtained by the naïve Bayes model. The AUC and accuracy metrics for logistic regression, gradient boosting, and random forest models are comparable. The ROC curves for each model demonstrate high and similar AUC values, indicating strong discriminative power and suggesting effective pattern learning from the data (**Figure 1**).

**Table 3.** Models Used in Analyses and Their Scores

	**Logistic Regression**	**Naïve Bayes**	**Gradient Boost**	**Random Forest**
AUC	82%	63%	81%	83%
Accuracy	89%	84%	89%	90%

Abbreviation: AUC, area under the curve.

For random forest, the best performing model, we determined that 194 true-positive patients were predicted to have NASH, and 7024 true-negative patients were predicted to be non-NASH. There were 70 false-positive (type I error) patients (ie, those who were predicted to have NASH but did not actually have it) and 769 false-negative (type II error) patients (ie, those who were not predicted to have NASH but did have it). In the error analysis of the random forest model, it was observed that only 9% of negative cases (type II errors) were incorrectly predicted, compared with 26% of positive cases (type I errors). This indicates a higher rate of false positives than false negatives. The prevalence of type II errors is particularly concerning as it signifies the model’s failure to identify existing cases, which could result in missed diagnoses and inadequate treatment. The model demonstrates better performance in minimizing type II errors than type I errors. **Table 4** shows the confusion matrix.

**Table 4.** Confusion Matrix and Risk Group with Potentials

**Predicted Condition**	**Actual Condition**		
	**NASH**	**Non-NASH**	**At Risk**
NASH	194 (TP)	70 (FP)	514 997 (12%)
Non-NASH	769 (FN)	7024 (TN)	3 668 015 (88%)

Abbreviations: FN, false-negative; NASH, nonalcoholic steatohepatitis; TP, true-positive.

The number of patients known to have NASH in the testing subset (769 false negatives + 194 true positives) was 963. Among the 4 183 012 patients who were previously assigned at-risk in the testing subset 514 997 patients (12%) were identified as being likely to have NASH, which is approximately 125 times higher than the number of patients in the study population who were identified as NASH-positive.

**Figure 2.**
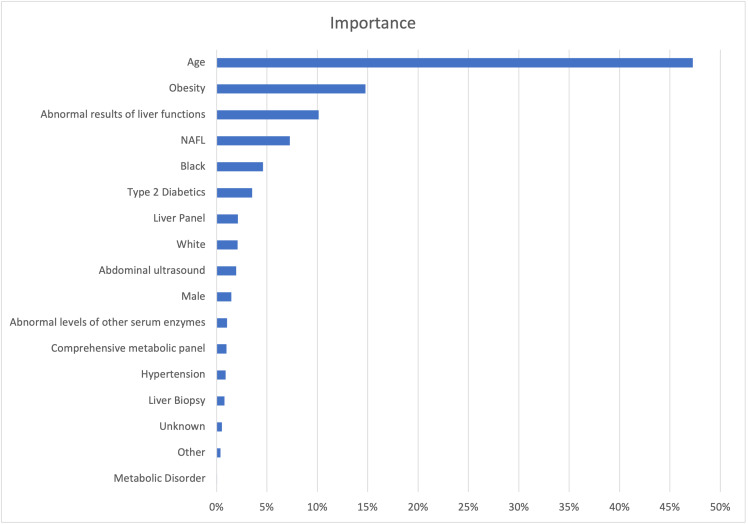
Feature Importance Measured by Contribution to Decrease in Gini Impurity Source: Data from present study. Abbreviation: NAFL, nonalcoholic fatty liver.

Using random forest to assess the impact of each feature on impurity, we found that age, obesity, and abnormal results of liver functions were the top 3 important determinants in assigning NASH probability. **Figure 2** summarizes the top 17 features that accounted for 90% of the model’s decrease in Gini impurity.

## DISCUSSION

In the wake of the increasing prevalence of NASH, the disproportionate increase in those with advanced fibrosis, liver failure, and hepatocellular carcinoma is concerning. In the United States, the prevalence of NASH is estimated at 20% to 30%^4^ and is currently the leading indicator for liver transplantation. Rises in NASH-associated liver cancer have been reported in US, Europe, and Asia.^23^ Moreover, NASH imposes a significant economic burden in the United States. In 2017, the lifetime costs of US NASH patients were estimated at $222.6 billion and the cost of the advanced NASH population at $95.4 billion.^24,25^

Since liver biopsy is not feasible in the general population, there are no studies that can accurately assess the incidence or prevalence of NASH.^24^ Some studies have indirectly assessed the prevalence of NASH and have indicated that the prevalence of NASH among NAFLD patients who underwent a random liver biopsy was 6.67%.^24^ The prevalence of NASH among NAFLD patients with a clinical indication for a liver biopsy was 59.10%.^24^ Given these estimates, the average prevalence of NASH was estimated to be between 1.5% and 6.45%.^24,25^

Furthermore, the prevalence of NASH has risen in parallel with obesity and diabetes, correlating with our results, which indicate 50.58% and 44.40% of the NASH population had been diagnosed with type 2 diabetes and obesity, respectively. Yasar et al^8^ showed that type 2 diabetes and obesity were more common in patients with NASH regardless of NAFL history. They stated that, although NAFL is a precursor of NASH, only 35% of patients with NASH received a NAFL diagnosis claim.^8^ In our study, only 15.32% (*P* < .0001) of the NASH population had an associated NAFL diagnosis.

Several studies have found a significant relationship between the number of metabolic syndrome components and the probability of NASH in patients with NAFLD.^26^ Our study supported this statement as it found that obesity, type 2 diabetes, and hypertension played a significant role. It indicated hypertension was a significant comorbidity in the NASH population (68.49%) and at-risk population (53.08%). This study supported other studies that have shown that risk factors such as advancing age and male gender play an important role in this disease. These risk factors have been used to create scores that predict NASH, such as an individualized polygenic risk score that considers sex, presence of metabolic syndrome, Gholam score (AST and DM), and other predictive models that include factors like hypertension and body mass.^26^

Despite the rising prevalence and serious potential clinical consequences of NASH, there are currently no treatments licensed for this disease. Since treatment relies on lifestyle changes, primarily weight loss, we found obesity to be the most significant clinical factor in our model to predict possible NASH patients from the at-risk pool.

The progression from simple hepatic steatosis to NASH is a crucial point in the development of severe liver diseases, putting patients at higher risk for fibrosis and progression to chronic liver disease.^8^ Still, NASH continues to be underdiagnosed in clinical practice due to unclear patient symptomatology and a lack of reliable identifiable biomarkers.^8,27,28^ Additionally, liver biopsy, the gold standard for NASH diagnosis, is costly and invasive, is complicated by sampling errors, and requires a specialist to perform.^8,29^ On March 14, 2024, the US Food and Drug Administration granted accelerated approval for resmetirom (Rezdiffra), the only current medication for the treatment of NASH.

Therefore, NASH underdiagnosis may prevent many patients from reaching these potential treatments. ML could potentially be used by providers or payers who wish to implement high-volume screening for suspected NASH in an at- risk patient population.^4^ ML using real-world data has been studied to help address the underdiagnosis of common and rare diseases. Despite the modest improvements observed in our study, where the random forest model increased the AUC from 82% to 83% and accuracy from 89% to 90%, ML models generally surpass conventional statistical methods. This superiority is attributed to their enhanced capability in identifying variables pertinent to clinical outcomes, superior predictive performance, adeptness at modeling complex relationships, ability to integrate multiple data modalities, and resilience to data noise.^15^ These small percentage increases can be particularly significant for life-threatening diseases such as NASH. Thus, noninvasive, cost-effective prediction models with good sensitivity and specificity are essential because, if NASH is detected early, treatment through lifestyle interventions can be effective.^5^

This study provides an approach to how ML can help to detect likely NASH patients from large at-risk patient populations using medical claims. The data-driven approaches are particularly valuable when diagnosis requires invasive or costly procedures or when the clinical risk factors that could screen patients are imprecise. NASH is difficult to detect without an invasive liver biopsy and is thus largely underdiagnosed despite the risk of progression to cirrhosis.

These models also identify patients who may benefit from clinical follow-up. For example, the likely NASH patients identified from our model can undergo elastography (Fibroscan®) for further clinical validation. Furthermore, the ML algorithm can be used to find both high-risk and low-risk subjects, who do not need further costly interventions. For example, 81.9% of unnecessary examinations in the United States could be avoided if a low-risk subject does not undergo abdominal ultrasound. Our research showed that 62.28% of ultrasound procedures performed on NASH patients could have been prevented and that only 31.53% of patients with NASH have abnormal liver function test results.

Our model identified 769 false-negative (type II error) patients and identified 514 997 patients (12%) as likely to have NASH, which is approximately 125 times higher than the number of patients in the study population who were identified as NASH-positive. In addition, our study identified the best predicting model was random forest. In assessing the impact of each feature on impurity using the random forest model, we found that age, obesity, and abnormal results of liver functions were the top 3 important determinants in assigning NASH probability. Several studies have supported age, obesity, abnormal liver functions as predictive parameters for NASH. NASH (steatohepatitis) is the driver of disease progression, whereas liver fibrosis is the link between liver injury and cirrhosis and its complications.^26^ Because liver biopsy is also a suboptimal reference standard with considerable sampling bias and intra-observer and inter-observer variability, there has been a surge in recent years in developing noninvasive tests for hepatic steatosis, steatohepatitis, and fibrosis.^26^

Patients with NASH have faster fibrosis progression and are at an increased risk of cirrhosis and hepatocellular carcinoma.^26,30^ Biomarkers like CK-18 fragments have been the most widely studied biomarker of NASH.^7,26^ Other biomarkers used for prediction include alanine aminotransferase^31^ levels; NASH score (PNPLA3 genotype, aspartate aminotransferase [AST], and fasting insulin); the NASH ClinLipMet Score (glutamate, isoleucine, glycine, lysophosphatidylcholine 16:0, phosphoethanolamine 40:6, AST, fasting insulin, and PNPLA3 genotype); individualized polygenic risk score (sex, presence of metabolic syndrome, homeostatic model assessment for insulin resistance, AST, PNPLA3, and HSD17B13 genotypes); and the NASH prothrombin time scoring system (PNPLA3 and TM6SF2 genotypes, diabetes status, insulin resistance, AST and high-sensitivity C-reactive protein).^26^

Several predictive models for the diagnosis of NASH combine clinical and laboratory parameters. The predictive models include hypertension; increased ALT; insulin resistance; Palekar score (age, sex, AST, body mass index, AST/ ALT ratio, and hyaluronic acid); Gholam score (AST and type 2 diabetes); oxNASH (13-hydroxyl-octadecadienoic acid/linoleic acid ratio, age, body mass index, and AST); NAFIC score (ferritin, insulin, and type IV collagen 7s); acNASH index (AST-to-creatinine ratio); and NashTest (Biopredictive); and a proprietary formula including 12 variables (age, sex, height, weight, serum levels of triglycerides, cholesterol, α_2_-macroglobulin, apolipoprotein A1, haptoglobin, GGT, aminotransferases ALT, AST, and total bilirubin).^26,32,33^ Additionally, some fibrosis markers like fibrosis-4 index and NAFLD fibrosis score are both cost-effective and sensitive panels to rule out patients with advanced fibrosis. Importantly, improvement in fibrosis with no worsening of NASH is another acceptable histologic endpoint for conditional approval for NASH drugs.^34^

Imaging studies like LiverMultiScan is a noninvasive, MRI-based biomarker to evaluate levels of liver fat, liver iron content, fibrosis, and inflammation.^26^ Magnetic resonance elastography examines the entire liver and has the advantages of less sample error, low failure rate, and high repeatability compared with biopsy procedures.^26^ However, these procedures are costly and not always available. Regarding liver fibrosis, liver stiffness measurement via transient elastography has high accuracy and is widely used across the world but may fail in some subjects like patients with obesity.^26^

Our model predicted that 26% of cases were falsely identified as having NASH, which they did not actually have. The financial implications of pursuing treatment for these false positives can be significant. Healthcare costs associated with NASH vary considerably, influenced by factors such as the presence of comorbidities like type 2 diabetes. For patients with NASH alone, the mean all-cause annual healthcare cost is approximately $7644, after excluding the top 1% of spenders to minimize data skew.^35^ This cost escalates markedly when NASH is accompanied by type 2 diabetes, with annual expenses reaching approximately $16 120.^18^ Moreover, confirmed NASH diagnosis in a patient’s medical record typically facilitates improved access to essential care and monitoring services. An increase in false positive NASH diagnoses could potentially lead to elevated health insurance premiums, not only for individuals with a NASH diagnosis but for the broader insured population. Consequently, to enhance the long-term validity of these predictive models, it is imperative to incorporate additional variables to reduce both false positives and false negatives.

Our research has the advantage of identifying at-risk populations through a cost-effective application of ML to help identify underdiagnosed and high-risk NASH patients for proper and timely treatment before it evolves into a higher, more costly disease burden to the patient and healthcare system. ML classifiers could help medical agencies achieve the early identification and classification of NASH, which is particularly useful for areas with poor economy, and the covariates’ importance degree will be helpful to the prevention and treatment of NASH.^15^

### Limitations

This study has several limitations related to the use of administrative data sets, which may be subject to inaccurate coding of patient clinical diagnoses and procedures, as well as clinical information limited to conditions and treatments defined by ICD-10-CM codes. Since the analysis was done on the review of claims data that were not originally designed for research, some information is bound to be missing. First, as with most claims-based data sources, there was a time lag between an individual’s receipt of services and when the files become available for research (average, 2-3 years). The data may not be generalizable to the entire population, as some information may be missed in processing or reimbursement. Additionally, not all health data were captured in the claims.

Although the VA data set was nationally representative, it was predominantly male and comprised a vulnerable population. Replication of this study using other data sets that are more representative in terms of gender and income distribution may be useful.

The ability of ML to predict NASH effectively is dependent on the quality of features within the model and the data used for training. The best set of features may also depend on the population under study as the incidence of NASH can vary across different ethnic groups.^1^ Finally, it is important to note that the correction models we applied have not been validated, and further research must be done in different settings.

## CONCLUSIONS

While biopsy is the reference standard for NASH diagnosis, reader agreement in interpretation of biopsies is a challenge, especially for less experienced readers^3^; further, it is an invasive and costly procedure. ML combined with digital pathology offers a possible solution to this problem. Studies have shown how ML allows quantitative analysis of steatosis, ballooning, inflammation, and fibrosis in biopsy samples to a high degree of accuracy.^3,31,36^

The utilization of ML to predict disease allows for wider recognition, timely intervention, and targeted treatments to improve or mitigate disease progression.^1^ Given that NASH is perceived as a silent and grossly underdiagnosed disease, this model could be utilized as an initial screening tool to select patients with potential NASH for further confirmatory diagnostic steps and clinical management. These findings should be further evaluated in prospective studies and since they use commonly available features, they could be automated through electronic health records systems and integrated into physicians’ workflows, leveraging laboratory tests already being performed.^1^
